# AI for Improving Children’s Health: A Community Case Study

**DOI:** 10.3389/frai.2020.544972

**Published:** 2021-01-06

**Authors:** Aakash Ganju, Srini Satyan, Vatsal Tanna, Sonia Rebecca Menezes

**Affiliations:** Saathealth, Mumbai, India

**Keywords:** low and middle income countries, digital health, artificial intelligence, health systems, machine learning

## Abstract

The Indian health care system lacks the infrastructure to meet the health care demands of the country. Physician and nurse availability is 30 and 50% below WHO recommendations, respectively, and has led to a steep imbalance between the demand for health care and the infrastructure available to support it. Among other concerns, India still struggles with challenges like undernutrition, with 38% of children under the age of five being underweight. Despite these challenges, technological advancements, mobile phone ubiquity and rising patient awareness offers a huge opportunity for artificial intelligence to enable efficient healthcare delivery, by improved targeting of constrained resources. The Saathealth mobile app provides low-middle income parents of young children nflwith interactive children’s health, nutrition and development content in the form of an entertaining video series, a gamified quiz journey and targeted notifications. The app iteratively evolves the user journey based on dynamic data and predictive algorithms, empowering a shift from reactive to proactive care. Saathealth users have registered over 500,000 sessions and over 200 million seconds on-app engagement over a year, comparing favorably with engagement on other digital health interventions in underserved communities. We have used valuable app analytics data and insights from our 45,000 users to build scalable, predictive models that were validated for specific use cases. Using the Random Forest model with heterogeneous data allowed us to predict user churn with a 93% accuracy. Predicting user lifetimes on the mobile app for preliminary insights gave us an RMSE of 25.09 days and an R2 value of 0.91, reflecting closely correlated predictions. These predictive algorithms allow us to incentivize users with optimized offers and omni-channel nudges, to increase engagement with content as well as other targeted online and offline behaviors. The algorithms also optimize the effectiveness of our intervention by augmenting personalized experiences and directing limited health resources toward populations that are most resistant to digital first interventions. These and similar AI powered algorithms will allow us to lengthen and deepen the lifetime relationship with our health consumers, making more of them effective, proactive participants in improving children’s health, nutrition and early cognitive development.

## Introduction

India’s urban population is expected to grow to over 550 million in the next decade, with nearly half the population residing in cities (State of the World Population, 2007). This trend represents an enormous opportunity for India’s growth and leadership in urban development, as well as a great challenge to deliver a higher quality of life to more citizens. The extent to which India’s health system can provide for this large and growing city-based population will determine the country’s success in improving the national health indices (Rao and Peters, 2015). As a country with a predominantly young population, a large share of the health system deficit threatens to impact young adults and their children. India is home to 472 million children under the age of 0–18 years, comprising 39% of the country’s total population. Out of the 128.5 million children residing in urban areas, close to 7.8 million children under the age of 0–6 years still live in poor conditions in informal settlements which are usually overcrowded, devoid of basic amenities and surrounded by a hazardous environment (NIUA, 2016).

Aggregate statistics show that on average, compared to their rural peers, urban children have access to better essential services such as health care and education, water and sanitation, and therefore better outcomes. A closer examination of the evidence, however, points to the fact that this urban advantage for children is perhaps an overgeneralization. Intra-urban disparities can be so large that many of the most disadvantaged children in urban areas fare worse than children in rural areas. In particular, the urban scenario of undernutrition confirms the hardships faced by poorer urban children. It is alarming that the percentage of underweight and stunted children in the age group of 0–59 months is highest among the urban poor, and is in fact higher than the overall rural figures (McKinsey Global Institute and Digital India, 2019). The lack of investment in public utilities coupled with substantial growth in the urban population due to a poverty-driven rural to urban migration, most urban dwellers have poor access to health and education services as well as inadequate availability of water and sanitation services. It is a matter of serious concern, as the first few years of a child’s life have a profound and enduring effect on the rest of the child’s life and, eventually, the lives of many others. Poverty, ill health, poor nutrition and a lack of stimulation during this crucial period can undermine educational foundations, restricting what children are able to accomplish (NIUA, 2016).

## Harnessing the Digital Revolution for Better Health

While health and nutrition outcomes depict one aspect of India’s development, the economy continues to grow, with industries such as telecommunications seeing an unprecedented growth in the last decade. India is estimated to host close to 560 million smartphone internet users (McKinsey Global Institute and Digital India, 2019). Monthly mobile data consumption per user is growing at 152% annually, more than twice the rates in the United States and China (McKinsey Global Institute and Digital India, 2019). Emerging internet users are primarily consuming the internet on their smartphones, and much of the new data consumption is driven by video in regional languages. The almost ubiquitous presence of mobile phones makes them a powerful channel for imparting health information to positively influence health seeking behavior in a cost-effective, scalable manner (Ganju et al., 2018). Our objective was to connect digital India with tools that could address the gaps in health awareness, nutrition practices and early childhood development.

### What We Do

Our digital platform “Saathealth” provides an ecosystem that connects underserved, low income parents with entertaining, gamified, behavior change content around four main themes–health, nutrition, early childhood development and learning. Our formative research began in the city of Mumbai, in Govandi - a community that records the lowest human development index in the city at 0.05, where more than half of the children studying in the local schools are malnourished (Social Economics Conditions and Vulnerabilities, 2015). By incorporating elements of gamification, the platform nudges parents toward healthy behavior change by shaping their understanding and perceptions.

The content on the Saathealth app was derived from an extensive literature review of global and local evidence on children’s health and nutrition issues. This evidence formed the basis of a weekly schedule of content, designed in multiple, consumable formats that include videos, infographics, notifications, and interactive quizzes. We gamified the user experience by tagging each video with a series of quiz questions, allowing the user to earn points. Users earned points for a variety of online activities that included video views, quiz responses and app sharing, all designed to promote on-app engagement with the critical behavior shift content pieces. The videos delivered health messages embedded in stories with a cast of characters, designed to build empathy for the users. The narratives used reflected the light-hearted nature of conversations, including subtle humor that one would see in many Indian families, delivered in Hindi, which is the language spoken by over half the country (Census of India, 2011). The content was initially tested in focus groups in Mumbai before being deployed on the app.

In 2019, the Saathealth experience was offered through an android mobile app that provided families access to engaging, scientifically credible behavior change communication. The content library is expanding daily, and currently holds over 1,000 pieces of nutrition, health and parenting content. Since its launch in 2018, the app has reached over 105,000 installs and has impacted over 54,000 underserved families across India. These families have cumulatively consumed over 2.25 million pieces of health, nutrition, parenting and early learning content, and are spread over five states in the country.

Innovative mobile health (mHealth) tools when contextualized to the local needs and deployed effectively provide a highly scalable opportunity to compensate for certain deficiencies of the healthcare workforce and infrastructure (PwC, 2017). The use of artificial intelligence, relatively nascent in resource-poor settings, holds tremendous promise to improve the productivity and efficiency of health services (Wahl et al., 2018). Machine learning, the most deployable artificial intelligence application, can be a catalyst to bridge the gap between an underserved population and an overburdened health system. Our team is building machine learning algorithms to predict, model and prevent user churn, with the intention of prolonging user engagement with our intervention, and to direct health resources toward users that are most in need for them.

## Methods

### Predicting User Churn and Days on App

We used machine learning to predict user churn, which is a metric used to quantify the number of users that have uninstalled an app in a pre-specified period of time. Churn analysis is the process through which actionable insights are applied to improve user retention. This involved preparing the selected data, data pre-processing, and transforming it into a form suitable for building machine learning models. Once the data is processed, the right methods to train are selected, fine-tuned, and the best performing models are applied to the data set. This process, known as classification, is a supervised learning approach in which the computer program learns from the data input in order to classify new observations. This was a binary classification problem, and the target variables for each user were either churn or non-churn.

Along with predicting user churn, we used the same dataset, data cleaning and procedure to predict the number of days a user will stay on the app before uninstalling, the target variable for which was days on app (DOA). For predicting DOA, the modeling was performed taking into account continuous data as the target. The regression methods that we ran were logistic regression decision tree regression, and random forest regression. The procedure for data extraction and cleaning were the same in these two predictions, except for the target variable being changed to a continuous variable, i.e., days on app.

### Optimizing Saathealth’s Data to Improve User Retention

The data generated by Saathealth users enabled us to use appropriate machine learning algorithms to ensure that the intervention learnt from, iterated and optimized based on user experiences. Through a live dashboard, data was captured from the activity performed by users that allowed us to monitor feedback and behavior. It was critical to monitor users’ activity and ensure that they completed a maximum number of days on the app. User data and analytics collected for this research was anonymized, and conducted under the agreement of Saathealth users via voluntary acceptance of terms and conditions as a prerequisite to joining the mobile app. The data, captured through analytics and the backend database, was collated before the stage of pre-processing.

### Data Cleaning and Determining Days on App

Data cleaning for all the incomplete features was done individually. In certain features, the incomplete values were assigned a value of “0” if they were not performed by the user, for example, in the case of “share app” clicks, videos count, quiz count, offers count; if the user had not performed these actions, the field would be empty and we assigned these a “0” value.

We determined the days on app (DOA) by subtracting the date of uninstall by the date of registration. By combining these two features, we calculated days on app, which was our target.

### Feature Engineering

In a high dimensional dataset, some attributes improve the measure of performance and are useful for decision-making while others are less important attributes. Feature engineering needs to be performed in order to select the features that contribute most to the prediction model. If there are irrelevant features in the dataset, the model learns from them and results in lower performance. Feature engineering enables the discovery of actionable insights when performed and executed effectively. The features we selected, indicated in Table 2, were chosen as a result of exclusion of certain features with little or no predictability of the target and by identifying highly correlated features. The features selected were user behavior features, describing how a user interacts with the app (e.g., session duration, number of times they open the app, videos watched, quiz questions answered, etc.).

**TABLE 1:** Important metrics to measure app engagement.

Metrics	Definition
Session	<10 s spent on the platform
Active user	User that has performed a session
Registered active user	User that has performed a session post registration
Daily active user	Users that log in on a daily basis
Monthly active user	Total number of users on the platform in a month
Days on app (DOA)	Number of days spent by an active user on an app before uninstall

**TABLE 2:** Features selected for prediction of user churn.

Features selected	Definition
EngagementTime	Time spent on the platform in seconds
SessionCount	Session defined at >10 s on platform
NotificationsReceived	Notifications sent to a mobile phone
NotificationsOpened	Notification banner clicked
NotificationsDismissed	Notification banner dismissed
ShareCount	Clicks on share button
QuizCount	Number of quizzes attempted
VideoCount	Number of videos watched
OffersCount	Clicks on nutrition product offers
TotalContent	Total number of quizzes and videos consumed
DaysOnApp	Number of days since installation

**TABLE 3:** Accuracy results of various machine learning models.

Model	RMSE (in days)
Logistic regression	76.47
Decision trees	30.13
Random forest	25.09

After the data was cleaned, it was split into the training set, which allows the system to learn, and the test set, which provides the evaluation of the model’s actual fit. Our dataset was split into the following: 70% training set and 30% test set.

### Testing Various Models to Predict User Churn on Saathealth

We attempted several models to evaluate the accuracy and efficiency with which we could get a prediction. Linear and logistic regression was selected for DOA and user churn evaluation, decision trees (classifier and regressor) were evaluated for improved accuracy compared to linear and logistic regression, and random forest was used to assess if we could obtain higher accuracy. We excluded gradient boosted regression algorithms due to the long training time and comparatively lower accuracy.

Finally, the following models were selected to generate insights on user churn and DOA:

Logistic regression: This is the most commonly used model when the data in use has binary output, so when it belongs to one class or another, or is either a 0 or 1, and when the value of the target variable is categorical in nature (Brownlee, 2016).

Decision Tree: This supervised learning algorithm splits a data sample into two or more similar sets based on the most important input variables for making a prediction. The aim of using a decision tree is to build a training model that can predict the target variable's class or value by learning basic rules obtained from the training data set (Predictive Analytics Made Easy, 2020).

Random Forest: This ensemble learning approach builds upon multiple decision trees to obtain a model with greater predictive consistency and accuracy. It constructs a large number of individual decision trees which are trained on varying subsets of input data. The output is calculated based on class predictions of each of the individual decision trees, or an average of the probability outputs from each tree. The final result is several individual decision trees, which act as a group, in which the classification performance is based on the majority votes (Criminisi et al., 2020; Sklearn Ensemble, 2020).

We ran the random forest model on a dataset from September 2018 to November 2019, and then repeated it on the dataset from November 2019 to February 2020 after actionable insights were made to improve retention of users.

## Results

### The Accuracy Obtained for the Various Models Was

The highest accuracy was obtained using the random forest model, since the aggregation of information from multiple uncorrelated trees in the model minimized the effect of individual errors, resulting in an algorithm that was resistant to overfitting. Through this model, we were able to determine the importance of each feature in relation to days on app (DOA). For the objective of retaining users on the app for as long as possible, this was a critical advantage through which we then examined which user activities had the most predictive power for determining churn or retention. Likewise, we could determine which points of intervention within the app could improve retention and engagement, resulting in greater engagement with the platform’s behavior change content.

### Impact of Predicting Churn and Days on App

Through generating a random forest model for the Saathealth dataset, we obtained a root-mean-squared error (RMSE) of 25.09. This is a measure of the performance of the model, and represents the difference between the predicted values and the actual values. The average number of days on app spent before a user would uninstall or churn was found to be 38 days for the time period of September 2018 to November 2019.

Through this analysis, we also had an indication of which features were important to determine which users were likely to churn and how each feature affected the probability of a user churning. The first evaluation of the Saathealth user dataset from September 2018 to November 2019 revealed that notifications opened had a 37% positive correlation with DOA. This indicated that notifications, when optimized, had the potential to improve retention of users on the app and decrease user churn. We began to adopt a deliberate notification strategy to improve the number of DOA spent by Saathealth users.

The strategies enlisted in Table 4 were implemented and evaluated on a weekly basis and a second churn analysis was performed as a follow up for the months of November 2019 to February 2020 using the same techniques. The positive correlation between notifications opened and DOA had increased to 48%, indicating that there were positive shifts in user churn due to a more effective and precise messaging strategy.

**TABLE 4:** Revamping the targeted messaging strategy to boost engagement and retention of families on the platform.

Messaging strategies adopted to decrease user churn
Targeted messaging to users who are minimally engaged (performs under 2 sessions/week)
Incentivizing users to increase engagement with content through points and rewards
Informing users of positive behavior change trends observed in the Saathealth community to increase motivation
Deployment of rich notifications that contain engaging images and text
Increasing the number of notifications from five per week to approximately 45–50 per week

When the same model that was trained using data from the time period of September 2018 to November 2019 was used to predict average DOA after the implementation of targeted messaging strategies (i.e., from November 2019 to February 2020), we found that the churn rate was significantly lower than the former predicted result. After taking into account the RMSE of 25.09, the predicted DOA was a cumulative average of 85 days, a 123.68% increase. This indicates early signs that the targeted interventions nudged users to staying on the platform for a longer duration, than was assumed based on predictive modeling from earlier users. The graph below (Figure 4), depicts the difference between the predicted DOA from the earlier time period that was used to train the model, and the actual DOA seen after improving the quality and quantity of targeted messaging.

**FIGURE 1:**
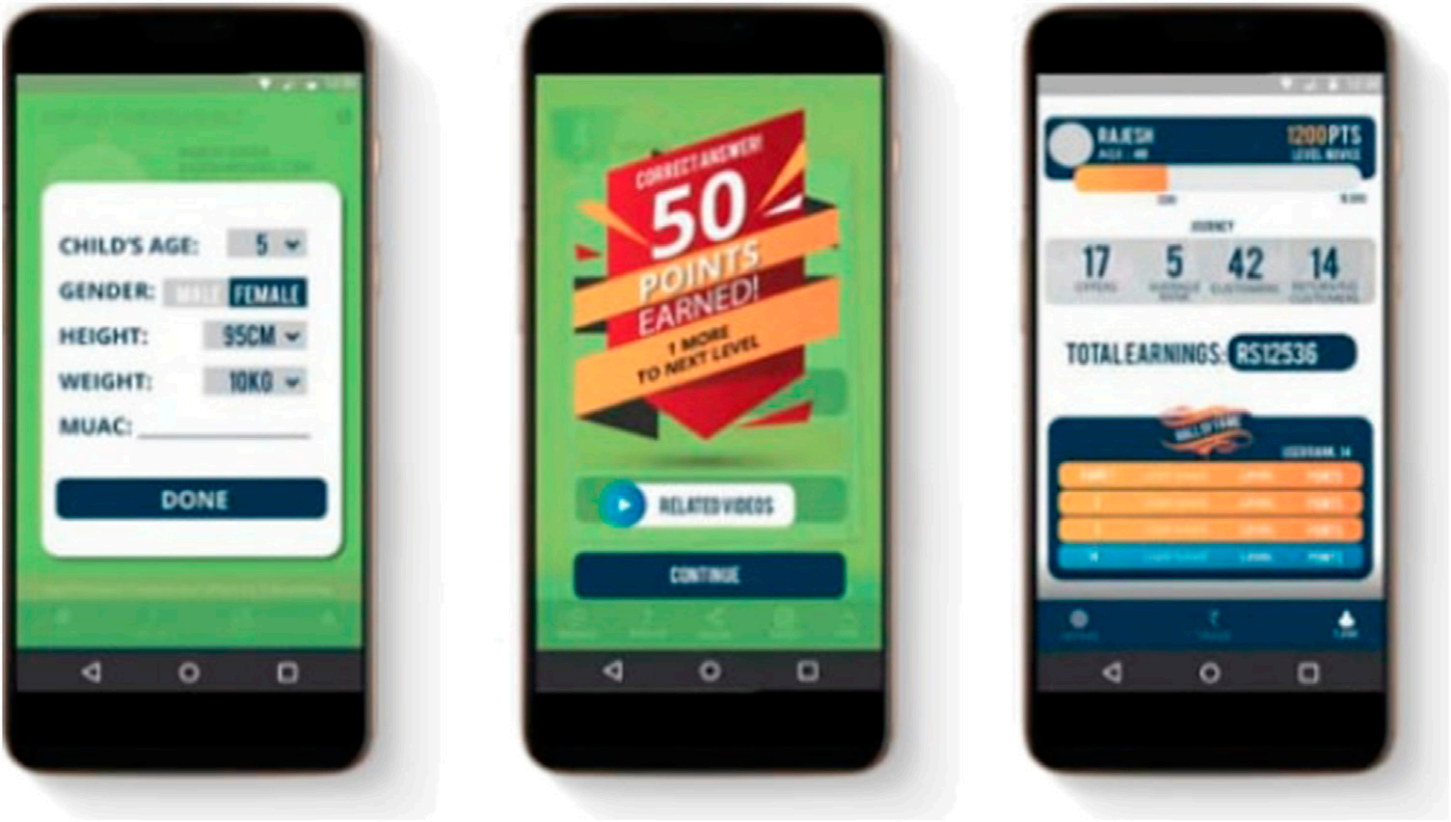
Screenshots of the Saathealth app.

**FIGURE 2:**
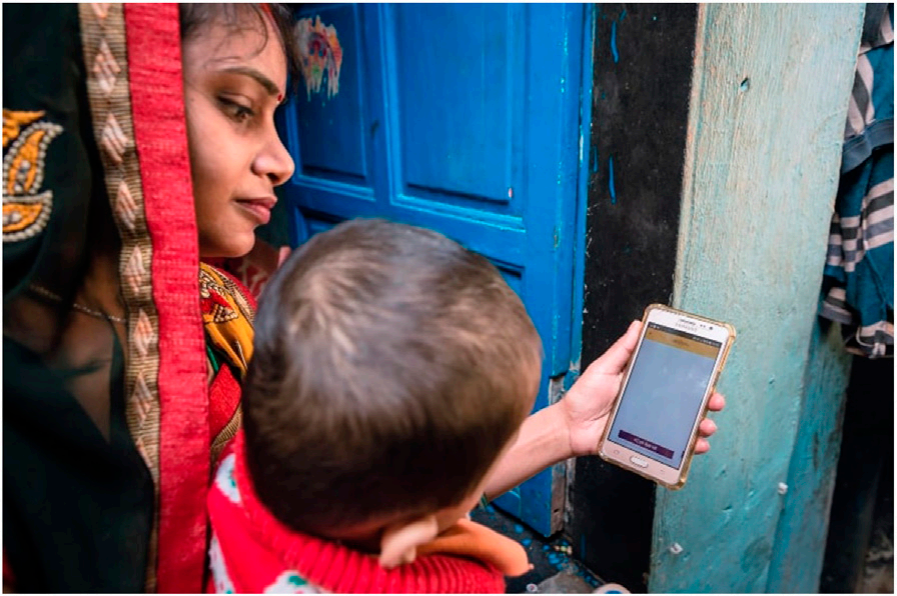
A mother using the Saathealth app.

**FIGURE 3:**
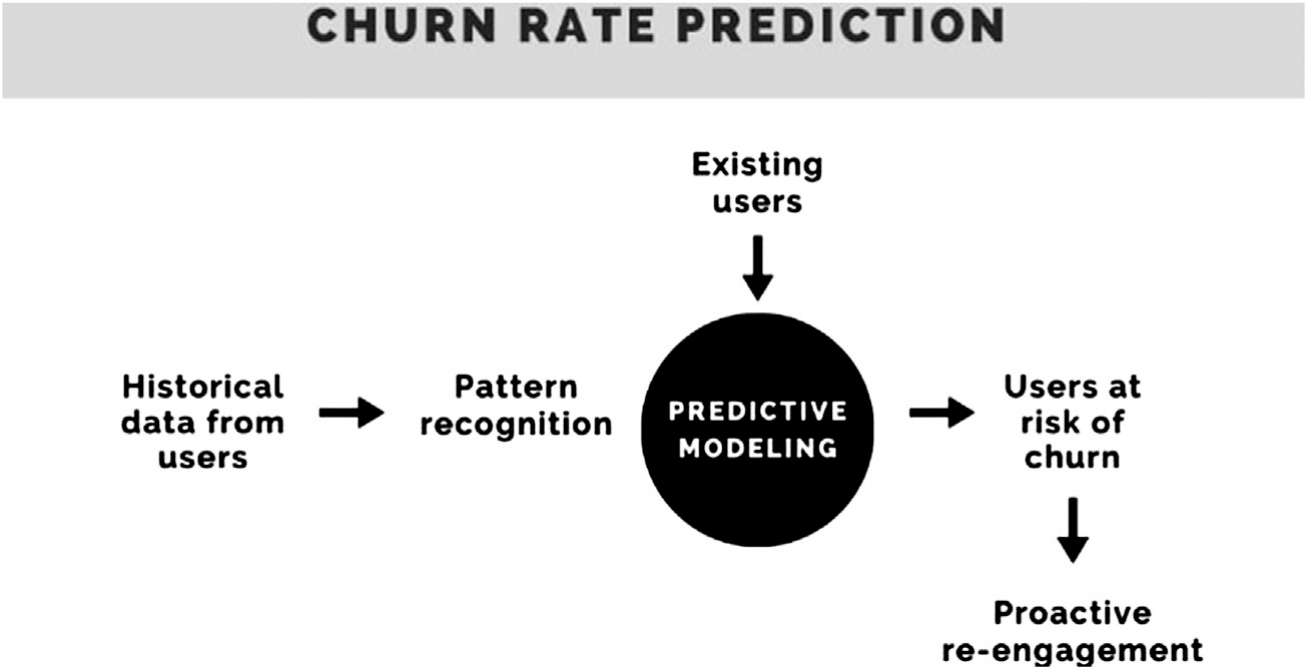
Prediction of user churn through machine learning modeling.

**FIGURE 4:**
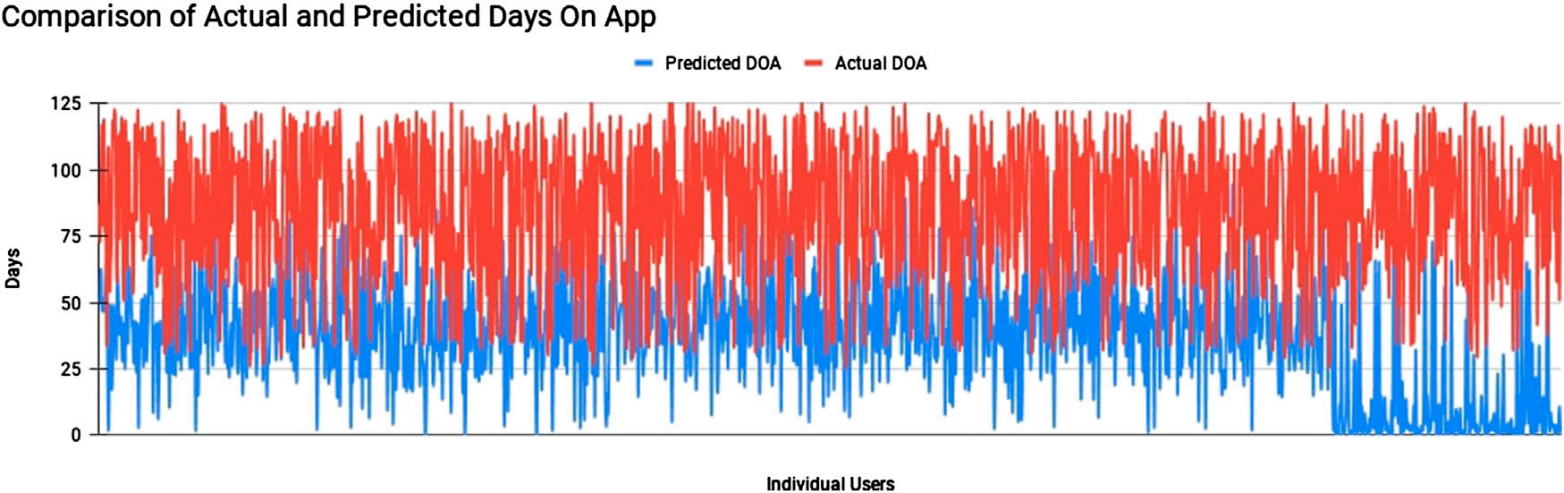
Comparison of DOA before and after revised targeted notifications strategy.

By effectively using machine learning models, we could identify the features that had the most significant impact on DOA and optimize those features to minimize user churn.

## Comparing Saathealth Retention With Other mHealth Platforms

The objective of maximizing the DOA was simple: engaging with users allowed us to incentivize positive health and nutrition behaviors, making parents active allies in improving their children’s health. We have a unique opportunity to engage families in underserved communities with scientifically credible, behavior change-driven content that was specifically designed to inspire parents with the aspirational yet attainable goal of a healthier future for their children. We succeeded in building a loyal user base that engaged consistently, and had higher retention than most other digital platforms. The average 30-day user retention for most health apps is less than 7%, conversely Saathealth users have a median 30-day retention of 18% (Neura, 2020).

While the last 10 years have spawned a wide number of mHealth initiatives, most of these are in high income countries with limited reported evidence of efficacy (Marcolino et al., 2018). Many mhealth initiatives have also reported limited uptake and engagement with patient groups (Kohl et al., 2013). The average app retains only 5% of its daily active users after 3 months, while 88% of Saathealth’s existing active users have been on the platform for a period of 3–5 months (Andrew, 2020). This opportunity allows us to reach millions of underserved Indians with essential health, nutrition and parenting information and incentives via a highly scalable, cost-effective digital platform. There have been numerous other digital interventions deployed as a part of government initiatives to equip frontline health workers to update their skills, stay in touch with supervisors, and track and report crucial data about health issues in their communities (IntraHealth, 2020). With platforms like Saathealth, we can meet the needs of health consumers that have been unresponsive to digital-first interventions. Similar apps and tools, in the hands of frontline health workers, can exponentially increase the effectiveness of awareness campaigns, with the ability to monitor engagement and rapidly iterate to meet the needs of the largest population of children globally.

## The Challenges and Opportunities in Low and Middle Income Countries

Low and middle income countries (LMICs) are increasingly having to combat the burden of a wide array of conditions like maternal and infant mortality, infectious diseases and chronic diseases. The deficit of health workers is adequately documented and while countries should continue to invest in expanding the number and the training of new health workers, it is equally imperative to start exploring the role of task shifting vital aspects of healthcare to the consumers. The expanding reach of mobile internet to low income populations offers a compelling opportunity to reach, educate and generate demand from health consumers. mHealth interventions have demonstrated value for patient care in a variety of scenarios (Rowland et al., 2020). Patient education interventions have already been shown to be an effective tool to cut healthcare costs by providing greater knowledge of illness, improves health related quality of life and self-management strategies, making patients informed, active allies in managing their own health (Stenberg et al., 2018).

The use of mobile apps has been shown to increase treatment adherence, and they are an appropriate method for managing medication at home (Pérez-Jover et al., 2019). However, the public health impact of mHealth interventions has often been limited by poor adherence, so predicting and increasing digital engagement of health consumers is important to enhance the effectiveness of such interventions (Carlo et al., 2019). Several other industries have already beneficially used the prediction of customer engagement, also known as churn prediction. Predicting engagement from user behavior provides the first critical step toward increasing the dosage, frequency and effectiveness of digital interventions. These prediction models can also be used to proactively direct resources toward settings at a higher risk of failure (Killian et al., 2019).

Our work with low-income communities in India offers an approach to predict and enhance digital engagement for public health improvements in children’s health and nutrition, especially in low-income communities. Our future work aims to focus on two main goals: to use our prediction models to enhance engagement with children’s health and nutrition consumers, and to demonstrate that user engagement prediction can increase frontline worker productivity by redirecting them toward higher burden health settings at risk of failure.

## Data Availability

The raw data supporting the conclusions of this article will be made available by the authors, without undue reservation.
